# Comparison of Gender Diversity Among Spine Surgeons in the Japanese Society for Spine Surgery and Related Research and the Neurospinal Society of Japan: A Descriptive Study Through Secondary Analysis of Aggregated Data

**DOI:** 10.7759/cureus.61152

**Published:** 2024-05-27

**Authors:** Tadatsugu Morimoto, Takaomi Kobayashi, Miyuki Fukuda, Hirohito Hirata, Koji Otani, Miho Sekiguchi, Kazuyo Yamauchi, Masatsugu Tsukamoto, Satomi Nagamine, Hirotaka Haro

**Affiliations:** 1 Orthopedic Surgery, Saga University, Saga, JPN; 2 Neurological Surgery, Shin-Aikai Spine Center, Katano Hospital, Katano, JPN; 3 Orthopaedic Surgery, Saga University, Saga, JPN; 4 Orthopedic Surgery, Fukushima Medical University, Fukushima, JPN; 5 Community-Oriented Medical Education, Graduate School of Medicine, Chiba University, Chiba, JPN; 6 Orthopedic Surgery, University of Yamanashi, Chuo, JPN

**Keywords:** neurospinal society of japan, japanese society for spine surgery and related research, spine surgeon, orthopedics, neurosurgery, gender diversity

## Abstract

Study design: This was a descriptive study through secondary analysis of aggregated data.

Purpose: This study aimed to describe changes in women's membership in the Japanese Society for Spine Surgery and Related Research (JSSR) for orthopedic surgery and the Neurospinal Society of Japan (NSJ) for neurosurgery over the past decade and make predictions for the future.

Overview of literature: Although the ratio of women physicians in the field of spine surgery is known to be low worldwide, there is a lack of detailed surveys in Japan.

Methods: We sent emails to the JSSR and NSJ secretariats to verify membership information (gender and age) from 2013 to 2022. Using ordinary least squares, we projected the years it would take for the JSSR and NSJ to achieve a gender diversity ratio of 30%.

Results: In 2013, the percentage of women in JSSR and NSJ was 2.3% and 2.7%, respectively. However, after 2018, the percentage of women in NSJ will be higher than in JSSR, rising to 2.7% in JSSR and 4.7% in NSJ by 2022. It would require 101 years for the NSJ and more than 1,000 years for the JSSR to realize 30% gender diversity.

Conclusions: JSSR and NSJ have low percentages of women. Improving gender diversity is an important issue for both societies, and they may collaborate on finding a good solution. Both the JSSR and NSJ societies need to actively address gender diversity and become more attractively represented in society for the next generation of spine surgeons.

## Introduction

In 2020, the women's physician ratio under the age of 30 in Japan was 36.2%, reflecting the recent increase in the number of women medical students [1,2]. On the other hand, the department with the lowest percentage of women in 2020 was that of orthopedics (5.7%), followed by neurosurgery (6.4%). These figures, unmistakably, did not align with the growing numbers of female physicians [3]. The low proportion of female orthopedic and neurosurgical surgeons remains a common problem in the USA, Europe, and Asia [4-6]. Worldwide, spine surgery is a subspecialty of orthopedics and neurosurgery. In Japan, similar to the global context, two major spine surgical societies exist: the Japanese Society for Spine Surgery and Related Research (JSSR) for orthopedic surgery and the Neurospinal Society of Japan (NSJ) for neurosurgery. The objective for enhancing gender diversity was to achieve a minimum of 30% representation of women [7]. The current rate of change from 2011 to 2022 was determined: it would take 160 years for the field of orthopedics and 135 years for neurosurgery to reach the target of 30% gender diversity (30% representation of women) [3]. Furthermore, in the 2020 population pyramid, men's orthopedic surgeons were most prevalent in the 50s, with declines in the 40s and 30s [3]. Given the trend away from surgery among younger male physicians and the increasing proportion of female doctors, specialties that have historically been unpopular with women are likely to face a downward trend in the future. This decline in these specialties could lead to a decline in the subspecialty of spine surgery. Although the prevalence of men in leadership roles within the fields of orthopedics and neurosurgery could be due to the limited representation of women, male physicians generally have a lower awareness of gender problems in the medical field [8,9]. Furthermore, many orthopedic and neurosurgical practitioners lack awareness regarding the current proportion of female spine surgeons. This information gap may exacerbate the existing situation.

In addition, the proportion of women physicians in orthopedics (2019) [10] and neurosurgery (2020) [6] by country in Asia is 10% and 16.3% in Malaysia, 5% and 5.3% in Indonesia, 3.8% and not stated in Thailand, 3.3% and 9.6% in the Republic of the Philippines, 3.2% and 4% in Singapore, Myanmar 2% and 3.5%, Sri Lanka 1.1% and 5%, Taiwan 1% and 3.8%, Korea 0.8% and 1.7%, India 0.5% and 2.8%, and China not stated and 2%. Thus, we suggest that these problems prevail not only in Japan but also in the Asian orthopedic and neurosurgical spine surgery societies.

Considering the strengths and challenges of diversity is critical. Meeting the diverse needs of diverse patients requires a diverse workforce with diverse values. In spine surgery, women physicians may be better able than men to gather information on genitourinary symptoms caused by spinal disease and to decide when to operate, taking into account the menstrual cycle and infertility treatment. Therefore, women physicians may be more satisfied with treatment than men physicians.

This study aimed to describe changes in women's membership in the JSSR and NSJ over the past decade and make predictions for the future. The differences and similarities in gender issues in the two societies obtained from this study will provide useful information for the resolution of this issue.

## Materials and methods

Since this was a de-identified, secondary analysis of aggregated data, no Ethics Committee approval was required. We emailed the JSSR and NSJ offices to confirm the membership data, encompassing the gender and age of their members, spanning 2013 to 2022.

Analyses

To quantitatively analyze the changes in women's membership in the JSSR and NSJ over the past decade, descriptive statistics for categorical data are presented as frequency (percentage). To compare qualitative variables between groups, the chi-square test was employed. To identify variations and shared characteristics in gender diversity across the two organizations, population pyramids were constructed for JSSR and NSJ members between 2013 and 2022. To predict the projected timeframe within which both societies could achieve the 30% gender diversity target, the study employed the ordinary least squares methodology. This method was used for estimating the duration required for JSSR and NSJ memberships to reach the specified goal. This forecast was derived from a straightforward linear regression analysis, which assessed the correlation between the number of years and the percentage of women. The percentage of women was analyzed as the dependent variable, while the number of years was considered the independent variable. The predictive models operated on the premise of an enduring linear trend, as affirmed by the data and the assumption of a sustained proportion of women [11].

The calculated regression coefficient, along with its associated 95% confidence intervals (CI), was considered the projected annual increase (PAN). Individual values of each of these, encompassing the regression coefficient and 95% CI, were considered indicative of the PAN for each successive year. The analytical procedures were performed with JMP® Pro software, version 16 (SAS Institute in Cary, NC, USA).

## Results

The changes in women's membership in the JSSR and NSJ over the past decade

Table 1 presents a comparison of gender ratios for JSSR and NSJ membership from 2013 to 2022.

**Table 1:** Comparison of gender ratios among members of the JSSR and NSJ membership from 2013 to 2022. JSSR: Japanese Society for Spine Surgery and Related Research; NSJ: Neurospinal Society of Japan. *Statistically significant.

		JSSR	NSJ	p-value
2013	Women	86 (2.3)	21 (2.7)	0.495
	Men	3658 (97.7)	755 (97.3)	
2014	Women	89 (2.4)	23 (2.8)	0.488
	Men	3635 (97.6)	797 (97.2)	
2015	Women	89 (2.4)	24 (2.8)	0.457
	Men	3682 (97.6)	835 (97.2)	
2016	Women	93 (2.4)	29 (3.2)	0.203
	Men	3725 (97.6)	883 (96.8)	
2017	Women	93 (2.4)	32 (3.3)	0.119
	Men	3722 (97.6)	927 (96.7)	
2018	Women	87 (2.3)	40 (3.9)	0.003*
	Men	3754 (97.7)	973 (96.1)	
2019	Women	87 (2.3)	44 (4.1)	0.001*
	Men	3740 (97.7)	1024 (95.9)	
2020	Women	87 (2.3)	51 (4.4)	<0.001*
	Men	3778 (97.7)	1107 (95.6)	
2021	Women	102 (2.6)	56 (4.5)	<0.001*
	Men	3843 (97.4)	1176 (95.5)	
2022	Women	108 (2.7)	62 (4.7)	<0.001*
	Men	3853 (97.3)	1244 (95.3)	

During this period, the number of men's memberships increased by 195 (from 3658 to 3853) and 489 (from 755 to 1244) in the JSSR and NSJ, respectively. However, the number of women's memberships increased by 22 (from 86 to 108) and 41 (from 21 to 62) in the JSSR and NSJ, respectively, with the number of NSJ women's memberships tripling during the past ten-year period.

**Figure 1:**
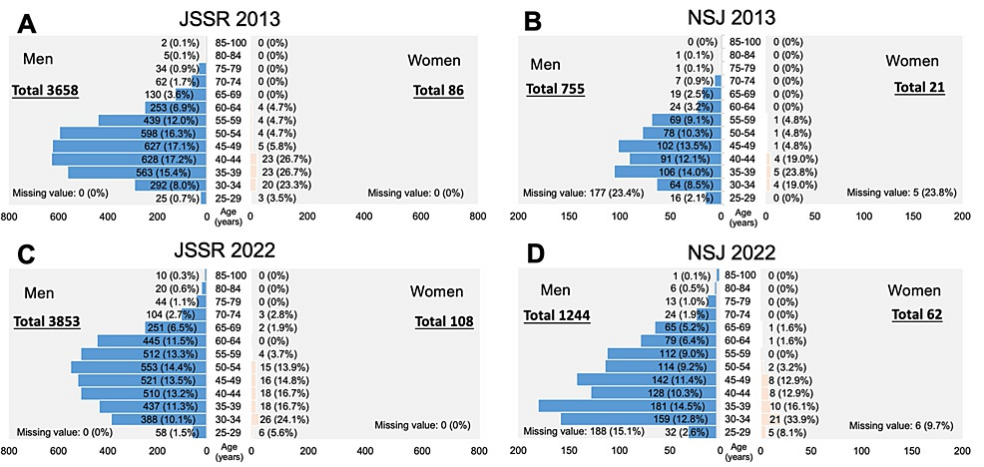
Demographic composition of orthopedic surgeons in the JSSR and NSJ by age and gender from 2013 to 2022. From 2013 to 2022, the largest age group of men moved from the 40s to the 50s, and the number of younger male spine surgeons declined. On the other hand, there has been a slight increase in the number of young women spine surgeons. JSSR: Japanese Society for Spine Surgery and Related Research; NSJ: Neurospinal Society of Japan.

In 2013, the percentage of women surgeons in the JSSR and NSJ was 2.3%. After 2018, the percentage of women surgeons in the NSJ exceeded that in the JSSR. By 2022, the percentage had increased to 2.7% in the JSSR and 4.7% in the NSJ. Figure 1 shows the demographics of the JSSR and NSJ memberships by age and gender in 2013 and 2022.

Some data regarding the age of NSJ were missing. In 2013, 23.4% (177/755 individuals) of men and 23.8% (5/21 individuals) of women were missing. In 2022, 15.1% (188/1244 individuals) of men and 9.7% (6/62 individuals) of women were missing. In 2013, the population pyramid for both the JSSR and NSJ showed that men's surgeons were mostly in their 40s, followed by those in their 50s and 30s. However, women were mostly in their 30s, followed by those in their 40s and 50s in both societies.

In 2022, the majority of men's surgeons in the JSSR were in their 50s, followed by those in their 40s, 30s, and 60s. However, most men's surgeons in the NSJ were in their 30s, followed by those in their 40s, 50s, and 60s. The largest numbers of women were in their 30s, followed by those in their 40s and 50s, in the same order as in 2013 in both societies.

When will JSSR and NSJ finally hit the 30% gender diversity goal?

The percentage in the JSSR will hit the 30% gender diversity goal by 3148, which will require 1126 years of history starting in 2022 (PAN: 0.024, 95% CI: −0.012 to 0.061) (Table 2). However, this is not statistically significant and is difficult to predict. Meanwhile, the NSJ will achieve the goal of 30% gender diversity in 2123, 101 years after 2022 (PAN: 0.25, 95% CI: 0.212% to 0.288%) (Table 2).

**Table 2:** Timeframe for JSSR and NSJ memberships to reach 30% of the gender diversity goal. JSSR: Japanese Society for Spine Surgery and Related Research; NSJ: Neurospinal Society of Japan; PAN: predicted annual increase; CI: confidence intervals.

	Estimated period (years)	PAN	95% CI	p-value
JSSR	1126	0.025%	−0.006% to 0.056%	0.100
NSJ	101	0.250%	0.212% to 0.288%	<0.001

## Discussion

This study aimed to identify the status of gender diversity in the JSSR and NSJ over the past 10 years.

Differences and similarities between the JSSR and NSJ in gender diversity and prospects

From 2013 to 2022, the ratio of women increased from 2.3% to 2.7% in the JSSR and from 2.7% to 4.7% in the NSJ. Based on the 2022 population pyramid, the number of young male spine surgeons was decreasing, but that of female surgeons was slightly increasing in the JSSR; meanwhile, both genders were increasing in the NSJ. A similar trend to the JSSR was shown in the population pyramid of the Japanese Orthopedic Association in 2020 [3]. Since JSSR is a subspecialty of the Japanese Orthopedic Association, it is natural that demographics would have an impact. According to the data released by the Ministry of Health, Labor, and Welfare, the ratio of women doctors in Japan increased from 19.7% in 2012 to 22.8% in 2020. However, in 2020, orthopedics had the lowest percentage of women surgeons in major medical specialties at 5.7%, followed by neurosurgery at 6.4% and cardiac surgery at 6.7% [3]. Furthermore, the JSSR and NSJ had lower percentages of women than orthopedics, indicating that improving gender diversity could be an important issue in both societies.

With regard to gender equality, the NSJ was more promising than the JSSR.

Regarding neurosurgeons, spine surgery generally has shorter operating times than neurosurgery, fewer emergency surgeries than stroke, and a higher quality of life. In addition, the physical fitness issue that has been a problem for women physicians has been overcome by the widespread use of minimally invasive spine surgery. These may explain why the number of NSJ spine surgeons has increased in both men and women. However, this finding needs to be verified in the future. Thus, the number of young spine surgeons in the NSJ has been increasing, resulting in an upward trend in the number of spine operations by Japanese neurosurgeons [12]. However, at the current pace, it will require 101 years for the NSJ and more than 1,000 years for the JSSR to realize 30% gender equality.

The number of spine surgeries has been increasing with the aging population [13]. Since spine surgeons in the 40-50 age group, the largest age group in the JSSR, retire, the number of spine surgeons may no longer meet the demand for spine surgery. The JSSR cannot neglect gender equality for over 1,000 years and the NSJ for 101 years. Thus, reducing gender disparity is a pressing priority for quality assurance in spine surgical care, not only to achieve gender diversity but also to preserve the number of spine surgeons.

Diversity in medicine: high value of women spine surgeons

Diversity in healthcare could facilitate better health outcomes, creativity, innovation, performance, and morale, enhance patient satisfaction and patient-centered communication, and further improve the aggregate knowledge and performance of the care team, ultimately directly benefiting both patients and surgeons [2,7,14-17]. Both fields of orthopedics and neurosurgery have low gender diversity. Reports on the reasons for low gender diversity have highlighted the disadvantages of women physicians, including a lack of role models and career obstacles due to life events such as pregnancy, childbirth, and childcare, as well as the physical challenges facing women physicians [3,15,18]. Therefore, there is a concern that the focus has not been on the advantages of women physicians in the spine surgery field. Although pregnancy may be a career barrier for female physicians, there may be advantages to being a physician who can relate to patients who have experienced pregnancy, childbirth, and childcare. Studies have demonstrated that patients often prefer to be treated by physicians of the same sex [19]. Intervention rates for orthopedic surgery are higher in women than in men [20].

Known common spinal disorders in women include adolescent idiopathic scoliosis [21], degenerative lumbar spondylolisthesis [22], and osteoporotic vertebral fractures [23]. Thus, the ideal situation might be where female patients could choose a female physician. Female patients may report less postoperative pain to male evaluators [19].

This reticence toward men may contribute to a failure to provide relief when a patient deviates slightly from the expected postoperative course. In cardiology and internal medicine, it has been implied that women may have a healthier prognosis when they are treated by female physicians [24]. Greenwood et al. demonstrated that in myocardial infarction cases, female patients treated by male physicians had a higher mortality rate. However, mortality rates were similar for men and women treated by female physicians [25]. Hospitalized older patients managed by female physicians have also shown lower mortality and readmission rates than those managed by male physicians [24]. These facts suggest that differences in practice patterns between men and women physicians may have critical clinical relevance to patient outcomes. Of note, women physicians understand more than men physicians the delicate issues that women patients face, such as urinary and genitourinary symptoms caused by spinal cord disease. Furthermore, they make surgical decisions that take into account the menstrual cycle and treatment plans that take infertility into account. In particular, in patients undergoing infertility treatment, fertility declines rapidly after the age of 35 [26]. Therefore, female physicians could make more appropriate decisions on the timing and content of treatment.

Proposed current and future efforts

The issues and causes of gender diversity are common in both societies. Regarding the impact of pregnancy, childbirth, and childcare life events on careers, both societies may benefit from the establishment of guidelines [10] that include institutionalized leave systems to assist life events such as childbirth, childcare, and family care.

Estonia (26.4%) and Sweden (16.8%), with the highest percentage of women orthopedic surgeons in 2020, have the most extensive parental leave and progressive social programs [5].

Social policies that support pregnancy and childcare, as well as academia, enable women's participation in the surgical workforce. Generous parental rights and progressive social policies may help promote women's participation in spine surgery. Poor work-life balance is another common problem. However, the issue of work-life balance is expected to improve in Japan with the introduction of working-hour regulations in 2024 and an improved environment.

Regarding the issue of physical strength, we suggest that with the development of minimally invasive spine surgery techniques and advances in robotic surgery, the number of surgeries requiring physical strength would decrease. Institutional leaders and senior mentors who support the academic activities of women spine surgeons may also be important in gaining gender diversity in academia [10,27]. Participation in academic meetings would also promote professional development, expand professional networks, and provide mentors and role models [27]. The problem of a lack of role models and mentors must also be resolved. The Gender Equality Bureau of Japan's Cabinet Office has recommended "positive action" for realizing a gender-equal society. Positive action refers to those taken to ensure that group members who have traditionally been treated unfairly for race, gender, or other reasons receive education, employment, and other benefits. Positive action has several methods that may be useful for JSSR and NSJ gender equality.

The most common approach is to set specific benchmarks for the recruitment of women to key positions in university hospitals and academic societies. However, there are few female mentors or role models in both societies. Therefore, a fellowship grant for aspiring women spine surgeons from both societies to a facility where women mentors and role models from both societies are in active practice could contribute to solving the problem. The issue of creating mentoring opportunities for women spine surgeons is a common problem in both societies, which could be resolved through collaboration between the two societies. Diversity generally attracts the best talent and leads to increased innovation. The JSSR and NSJ need to actively address gender diversity and become more attractive to the next generation of spine surgeons. Although diversity-informed strategies vary by country, the principles would apply not only to Japan but also to other Asian countries.

There are several limitations to the study. The presence of numerous missing values pertaining to the age of NSJ membership posed challenges in the evaluation process. The absence of this critical data point makes it intricate to accurately assess and analyze the demographics of the NSJ membership. The second limitation is that this study only ascertained the current situation through direct electronic methods (e-mail) to the society's office. A direct questionnaire to women spine surgeons is the next task, which would highlight the problems and solutions to the shortage of women spine surgeons. Finally, the worldwide percentage of women physicians in spine surgery may be low, but there are no detailed study cases available. This is also a future challenge.

Comparing countries with low and high numbers of female spine surgeons may provide pertinent results and indicate solutions regarding the role of government in accepting specialists and more privileges (e.g., salary) through gender-based capacity decisions.

## Conclusions

The gender demographic trends of the JSSR and NSJ over the past 10 years were described. From 2013 to 2022, the proportion of women increased slightly in both the JSSR and the NSJ. Both societies lag in gender diversity. Moreover, according to the current annual rate of change in this survey, it will take many years for both the JSSR and the NSJ to reach 30% gender diversity. Both the JSSR and NSJ may become more attractive to the next generation of spine surgeons by actively and cooperatively addressing the common issue of gender diversity.

## References

[1] (2024). e-Stat MoH, Labor and Welfare: Statistics on physicians, dentists, and pharmacists. https://www.e-stat.go.jp/stat-search.

[2] Niizeki Yumi KY, Emiko H (2022). Gender diversity in Japanese Orthopaedic Surgeons; efforts of the Japanese Orthopaedic Association. Orthop Surg Traumatol.

[3] Morimoto T, Kobayashi T, Yamauchi K (2023). How long will it take to reach the gender diversity goal for orthopaedics in Japan?. J Orthop Sci.

[4] Garozzo D, Rispoli R, Graziano F (2022). Women in neurosurgery: historical path to self-segregation and proposal for an integrated future. Front Surg.

[5] (2020). Diversity in orthopaedics and traumatology: a global perspective. EFORT Open Rev.

[6] Drummond KJ, Kim EE, Apuahe E (2021). Progress in neurosurgery: contributions of women neurosurgeons in Asia and Australasia. J Clin Neurosci.

[7] Harrington MA, Rankin EA, Ladd AL, Mason BS (2019). The orthopaedic workforce is not as diverse as the population it serves: where are the minorities and the women?: AOA critical issues symposium. J Bone Joint Surg Am.

[8] Rosta J, Aasland OG (2014). Weekly working hours for Norwegian hospital doctors since 1994 with special attention to postgraduate training, work-home balance and the European working time directive: a panel study. BMJ Open.

[9] Sexton KW, Hocking KM, Wise E (2012). Women in academic surgery: the pipeline is busted. J Surg Educ.

[10] Green J, Chye VP, Hiemstra LA, Felländer-Tsai L (2020). Diversity: Women in orthopaedic surgery—a perspective from the International Orthopaedic Diversity Alliance. J Orthop Trauma.

[11] Acuña AJ, Sato EH, Jella TK, Samuel LT, Jeong SH, Chen AF, Kamath AF (2021). How long will it take to reach gender parity in orthopaedic surgery in the United States? An analysis of the National Provider Identifier Registry. Clin Orthop Relat Res.

[12] Shouda M (2013). Report on National Statistical Survey on spine surgery in neurosurgical facilities. Spinal Surg.

[13] Imajo Y, Taguchi T, Yone K (2015). Japanese 2011 nationwide survey on complications from spine surgery. J Orthop Sci.

[14] Bennett CL, Baker O, Rangel EL, Marsh RH (2020). The gender gap in surgical residencies. JAMA Surg.

[15] Tougas C, Valtanen R, Bajwa A, Beck JJ (2020). Gender of presenters at orthopaedic meetings reflects gender diversity of society membership. J Orthop.

[16] Dineen HA, Patterson JM, Eskildsen SM, Gan ZS, Li Q, Patterson BC, Draeger RW (2019). Gender preferences of patients when selecting orthopaedic providers. Iowa Orthop J.

[17] Roter DL, Hall JA, Aoki Y (2002). Physician gender effects in medical communication: a meta-analytic review. JAMA.

[18] Johnson GW, Almgren-Bell A, Skidmore A (2022). Representation of female neurosurgeons as abstract authors at neurological surgery conferences. J Neurosurg.

[19] Wallis CJ, Jerath A, Coburn N (2022). Association of surgeon-patient sex concordance with postoperative outcomes. JAMA Surg.

[20] Borkhoff CM, Hawker GA, Wright JG (2011). Patient gender affects the referral and recommendation for total joint arthroplasty. Clin Orthop Relat Res.

[21] Weinstein SL (2019). The natural history of adolescent idiopathic scoliosis. J Pediatr Orthop.

[22] Kalichman L, Kim DH, Li L, Guermazi A, Berkin V, Hunter DJ (2009). Spondylolysis and spondylolisthesis: prevalence and association with low back pain in the adult community-based population. Spine (Phila Pa 1976).

[23] Hagino H (2021). Current and future burden of hip and vertebral fractures in Asia. Yonago Acta Med.

[24] Tsugawa Y, Jena AB, Figueroa JF, Orav EJ, Blumenthal DM, Jha AK (2017). Comparison of hospital mortality and readmission rates for Medicare patients treated by male vs female physicians. JAMA Intern Med.

[25] Greenwood BN, Carnahan S, Huang L (2018). Patient-physician gender concordance and increased mortality among female heart attack patients. Proc Natl Acad Sci U S A.

[26] von Wolff M, Schwartz AK, Bitterlich N, Stute P, Fäh M (2019). Only women's age and the duration of infertility are the prognostic factors for the success rate of natural cycle IVF. Arch Gynecol Obstet.

[27] Morimoto T, Kobayashi T, Yamauchi K (2024). Gender diversity of the Japanese Society for spine surgery and related research annual meetings from 2013 to 2022. Spine Surg Relat Res.

